# Hypertonic Saline in Treatment of Pulmonary Disease in Cystic Fibrosis

**DOI:** 10.1100/2012/465230

**Published:** 2012-05-03

**Authors:** Emer P. Reeves, Kevin Molloy, Kerstin Pohl, Noel G. McElvaney

**Affiliations:** Respiratory Research Division, Department of Medicine, Royal College of Surgeons in Ireland, Education and Research Centre, Beaumont Hospital, Dublin 9, Ireland

## Abstract

The pathogenesis of lung disease in cystic fibrosis is characterised by decreased airway surface liquid volume and subsequent failure of normal mucociliary clearance. Mucus within the cystic fibrosis airways is enriched in negatively charged matrices composed of DNA released from colonizing bacteria or inflammatory cells, as well as F-actin and elevated concentrations of anionic glycosaminoglycans. Therapies acting against airway mucus in cystic fibrosis include aerosolized hypertonic saline. It has been shown that hypertonic saline possesses mucolytic properties and aids mucociliary clearance by restoring the liquid layer lining the airways. However, recent clinical and bench-top studies are beginning to broaden our view on the beneficial effects of hypertonic saline, which now extend to include anti-infective as well as anti-inflammatory properties. This review aims to discuss the described therapeutic benefits of hypertonic saline and specifically to identify novel models of hypertonic saline action independent of airway hydration.

## 1. Introduction

**Figure 1:**
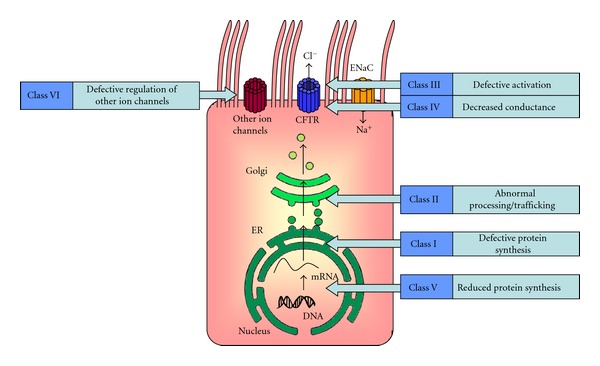
Classification of CFTR mutations. CFTR mutations are classified into six classes according to their effect on CFTR function. Class I mutations inhibit biosynthesis, while Class II mutations affect protein processing. Milder mutations such as Class III, IV, and VI impair CFTR channel function and Class V mutations affect gene expression, adapted from Allen (1999) [8].

Cystic fibrosis (CF) is a complex genetic disease with protean manifestations, the most important being increased risk of chronic lung disease resulting in terminal respiratory failure [1]. CF is an autosomal recessive disorder caused by mutations in the CF transmembrane conductance regulator (CFTR) chloride channel. More than 1000 mutations in the CFTR gene have been identified and result in misfolding of the CFTR protein. Reported mutations can be broadly categorised by class mutations which alter CFTR processing (Classes I, II, and V) as well as those resulting from dysregulated chloride secretion (Classes III, IV, and VI) (Figure 1). The commonest genetic defect in CFTR processing includes the ΔF508 mutation, of which 90% of CF suffers carry one copy [2]. Defects in CFTR protein function not only impact upon cAMP-dependent chloride secretion but also result in increased epithelial sodium channel- (ENaC-) mediated ion absorption in the superficial airway epithelium [3, 4]. As a consequence, increased water reabsorption across airway epithelial cells leads to extreme dehydration of the airway surface liquid layer, chronic mucostasis, and airflow obstruction [5]. This thickened mucus provides an ideal environment for bacterial infection in the respiratory tract with *Staphylococcus aureus* (*S. aureus*) being a major bacterial pathogen in early years and *Pseudomonas aeruginosa* (*P. aeruginosa*) a prominent pathogen in adult patients [6, 7].

Dehydration of the airway surface liquid layer has been implicated as the primary initiating event in CF-related lung disease [9] and therapeutic interventions to improve mucus clearance is a cornerstone of treatment in CF [10]. Such interventions include regular chest physiotherapy, mucolytics such as dornase-alpha (DNase) [11] and also aerosolized hypertonic saline (HTS; 3% to 7% NaCl) [12]. HTS is defined as a solution possessing an osmotic pressure greater than that of physiologic isotonic salt solution (0.9% NaCl). Inhalation of HTS has been proposed to significantly improve mucociliary clearance [13] and the popularity of its use has increased on the basis of a number of clinical trials [14–17]. Several mechanisms have been proposed for the observed effectiveness including changes in the rheological characteristics of the airway mucus [18], increasing airway surface liquid hydration [19], inhibition of ENaC [20], as well as immunomodulatory effects [21–23]. While a large controlled study reported mild positive effects of HTS on lung function [24], further studies have hurled it back into the limelight [25] and it is in this context that this review will compile the evidence for the use of HTS in treatment of individuals with CF.

## 2. Physical Properties of Airway Mucus in Cystic Fibrosis

**Figure 2:**
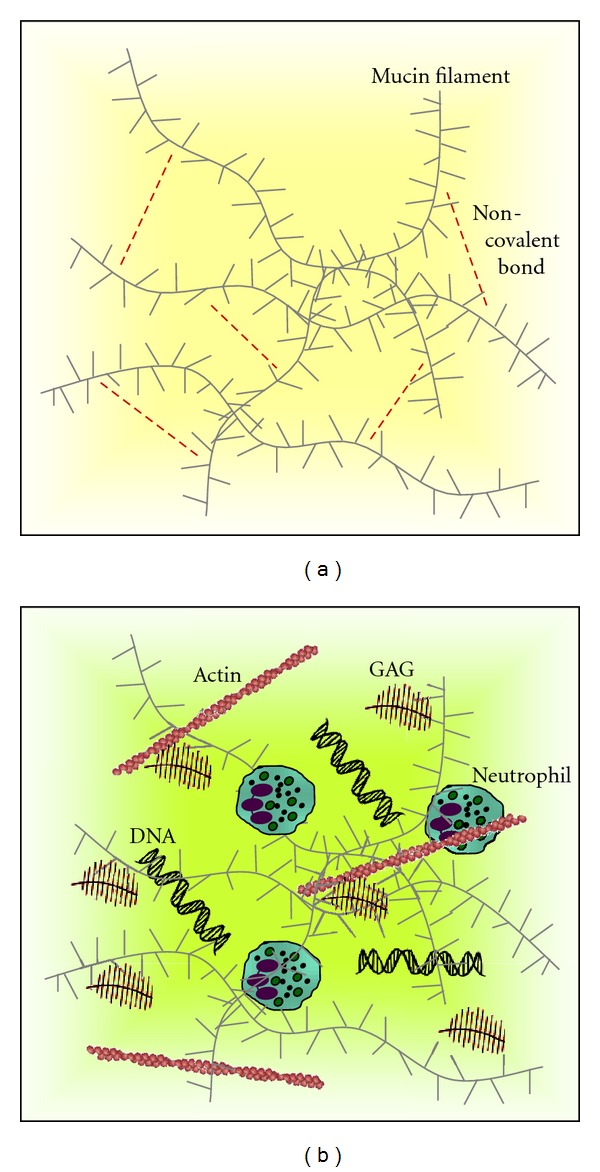
Mucus properties in the CF lung. (a) Mucus in a healthy lung is made up of a network of mucin filaments consisting of highly glycosylated mucin monomers that are crosslinked by disulphide bonds. Mucin filaments are bound together by noncovalent bonds (red dotted lines) such as van der Waals forces. (b) In the CF airways, mucus viscosity is increased by DNA and actin (red) that are released from necrotic neutrophils and aggregate into bundles. Glycosaminoglycans (GAGs, depicted in brown) which are important for regulation of cell interactions have been found to be upregulated and altered in CF. Adapted from Rogers (2007) [26].

**Figure 3:**
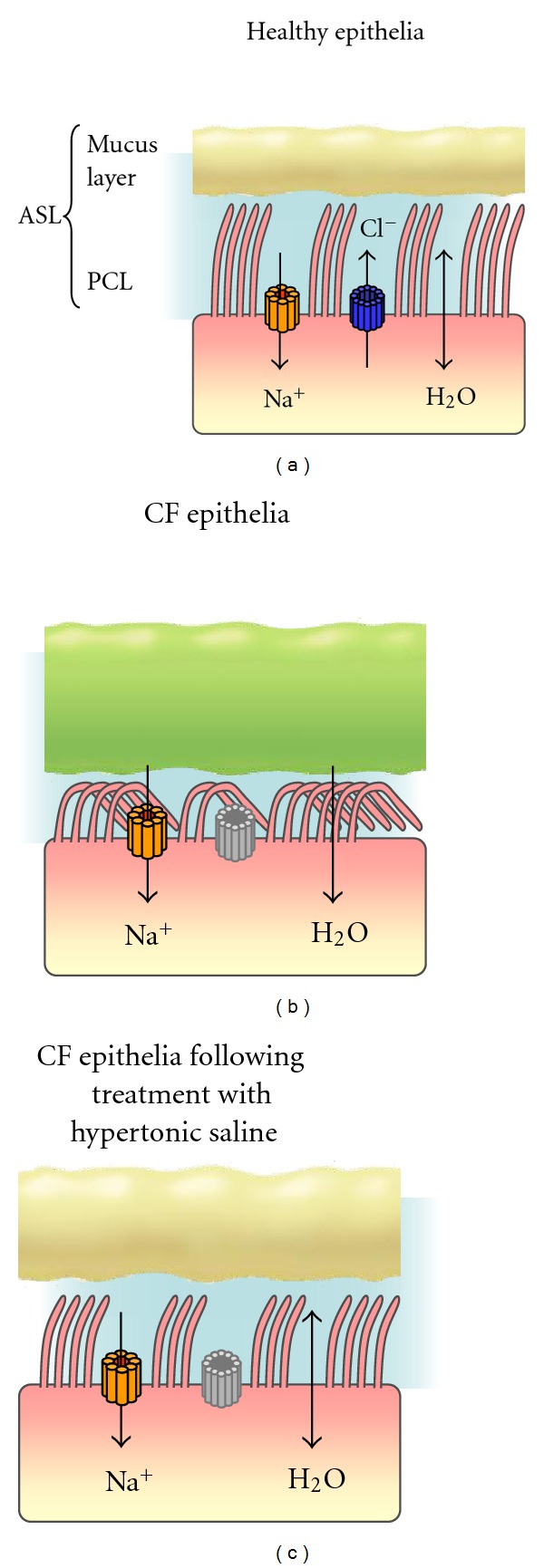
Effect of hypertonic saline on the airway surface liquid (ASL) in CF. (a) In healthy airway epithelia, CFTR is intact and plays a vital role in regulating hydration of the ASL that consists of the periciliary layer (PCL) and the mucus layer. (b) Due to defective CFTR in CF, Cl^−^ secretion is impaired and Na^+^ absorption through ENaC is upregulated resulting in dehydration of the ASL with thick mucus accumulating and causing the PCL to collapse. (c) Treatment with hypertonic saline is proposed to reduce mucus viscosity and aids its clearance by various mechanisms. The high salt concentration encourages osmosis of water into the ASL and thereby rehydrates the mucus and partially restores the PCL allowing for easier clearance of mucus. Additionally, the high ionic strength weakens ionic bonds between negatively charged GAGs and thus reduces the viscosity of the mucus.

Within the normal lung, the mucous gel is largely made up of mucin glycoproteins which are either secreted or cell membrane tethered. Airway epithelial cells express three gel-forming mucins including MUC2, MUC5AC, and MUC5B, although MUC5AC and MUC5B are thought to be the major gel-forming mucins in healthy airway secretions. While the molecular mass of mucins in CF and healthy control airway samples is comparable, the concentration of MUC5AC and MUC5B in CF sputum is markedly decreased [27]. This is of major importance and has prompted research aimed at investigating both the cause-and-effect relationship leading to thick purulent mucus in the CF airways. The primary cause of dehydrated thick mucus is increased water reabsorption across CFTR defective airway epithelial cells. However, a second reason for the thick viscous mucus in CF is the enrichment of anionic polyelectrolytes including DNA produced by colonizing bacteria or released from lysed inflammatory cells [28, 29]. Moreover, F-actin released from necrotic cells within the CF airways plays a major role in the secondary polymer network of CF sputum [30]. By laser scanning confocal microscopy, copolymers of DNA and F-actin have been observed which are thought to influence the viscoelasticity of CF sputa [31, 32]. In addition, elevated concentrations of anionic glycosaminoglycans (GAGs) have been found in mucus samples from children with CF [33] (Figure 2). For example, significantly increased bronchial levels of hyaluronic acid have been reported [34], with sputum concentrations 100-fold higher than in acute bronchitis. Moreover, CF sputum has been shown to contain significantly elevated levels of chondroitin [35] and heparan sulphate [36] and *in vitro* enzymatic digestion of GAGs with chondroitinase ABC rather than protein digestion with trypsin decreased viscoelasticity of CF purulent sputa [37]. Of importance, studies have also shown that mutations in CFTR give rise to aberrant levels of sulphation. Particular changes in GAG chains and sulphation patterns may allow increased interactions, normally of ionic nature, with various proteins including antimicrobial peptides [21] and proinflammatory stimuli [36, 38–40]. Data supporting this phenomenon have demonstrated synthesises of oversulphated glycoconjugates by CF tissue in organ culture [41] and airway epithelial cells [42, 43]. Fundamentally, regardless of the exact or combined cause of increased viscosity, the thickened mucus within the CF airways becomes detached from the cilia and mucociliary transport is impaired, the major cause of lung morbidity and mortality in CF. HTS is thought to act by drawing water from CFTR defective airway epithelial cells, thereby rehydrating the periciliary layer [44] and supporting mucociliary clearance [25] (Figure 3). HTS is, therefore, one of the first treatments whose mechanism of action bypasses the basic CFTR defect.

## 3. Hypertonic Saline in Treatment of CF Airway Disease

In 2006, Elkins and colleagues from the National Hypertonic Saline in Cystic Fibrosis Study Group performed a trial of 164 patients who were randomised to receive 7% HTS or isotonic 0.9% saline. Results revealed no significant difference in the rate of change of lung function (as measured by forced vital capacity and forced expiratory volume in one second (FEV1)), yet the absolute difference in lung function between groups was significant (*P* = 0.03). Of major importance, this study offered the first evidence for the long-term efficacy of HTS and showed a marked reduction in the number of exacerbations over patients who received isotonic saline solution [24]. Likewise, in a study performed by Donaldson and colleagues (2006), patients who received HTS (7% four times daily for 14 days with or without amiloride (inhibitor of ENaC)) demonstrated improved mucociliary clearance and FEV1 when given alone rather than in conjugation with amiloride [25]. The effect of HTS in this latter study could only be compared to baseline as no isotonic saline control group was included [25], however, their conclusions were echoed in a further study by use of radioaerosol technology [13]. Within this latter study, patients with CF treated with HTS had significantly increased mucociliary clearance compared to patients treated with amiloride or isotonic saline [13]. A further consideration when debating the effectiveness of HTS is the volume administered. In studies performed by Elkins et al., [24] and Donaldson et al., [25] 4 and 5 mL of HTS were aerosolized, respectively, and their studies recorded smaller improvements in lung function compared to Ballmann and von der Hardt [15] and Eng et al., [16] who delivered 10 mL of HTS. Moreover, in a recent stratified assessment of the impact of HTS on CF pulmonary exacerbations, Dmello et al., (2011) reviewed 340 cases of which 99 involved HTS treatment. By the use of multivariate logistic regression analysis, their results supported treatment with HTS in the context of reducing pulmonary exacerbations associated with CF lung disease [45].

HTS has been shown to increase mucociliary clearance in adults with CF [13, 25, 44], but in contrast, did not significantly improve mucociliary clearance in children with CF (median age 10.5 years) [46]. The differences in therapeutic efficacy of HTS between adults and children may, however, relate to the degree of airway disease. Nevertheless, if HTS can improve mucociliary clearance by impacting upon the fundamental hydration defect, it may prove most successful in treatment of juvenile and young teenage patients before airway disease takes hold. To this end, in a recent study of 18 young participants (12–30 months of age) who received 7% HTS twice daily for 14 days, Rosenfeld and coworkers (2011) showed that HTS was well tolerated with high patient adherence [47]. In addition, lung clearance index, a measure of lung physiology derived from multiple breath washout tests, improved in paediatric patients (6–18 years) treated with 7% HTS compared to isotonic saline (0.9%) solution [14]. Furthermore, a study assessing the safety and tolerability of inhaled HTS pretreated with nebulised beta adrenergic agonists (salbutamol) in preschool children (mean age 5.7 ± 0.8 years) and infants (mean age 1.6 ± 1 years) with CF showed no clinically important decrease in FEV1, FVC, or FEF_25–75_ after HTS treatment. Only one patient had a clinically significant decrease (>20%) in pulmonary function, however, this patient had a preexisting diagnosis of bronchial hyperactivity [48].

Induced cough may also be an important mechanism by which HTS improves mucociliary clearance in patients with CF. Patients treated with HTS have more cough episodes following treatment [48] and this may in itself improve mucociliary clearance by generating high sheer stress which promotes clearance of mucus from the airway surface [49]. The optimal dose of HTS is still a matter of debate with increasing doses (from 3% to 7%) requiring increasing nebulisation times, a potentially unrealistic treatment burden on patients [50], with compliance reported at only 64% [24]. On this point, coaerosolisation of 7% HTS with 0.1% hyaluronate has been shown to significantly improve tolerability and pleasantness compared to HTS alone [51]. Moreover, the effect of HTS on mucociliary clearance appears dose dependent. In a study performed by Robinson and colleagues (1997), the levels of sputum cleared over 90 minutes increased as the concentration of NaCl incrementally increased (0.9%, 3%, 7%, and 12%). However, patients experience considerable oropharyngeal irritation with concentrations approaching 12% making this the upper limit of tolerability in most patients.

 Improvements in mucociliary clearance by HTS may provide a useful adjunct to improve the amount of sputum available for microbiological evaluation from patients such as those with mild disease or young children who have difficulty expectorating sputum. These patients often undergo flexible bronchoscopy and bronchoalveolar lavage to obtain adequate sample volumes for culture and sensitivity. Thus, HTS-induced sputum may be clinically useful by increasing the volume of expectorated sputum available for microbiological culture [52], as previously shown for children with CF [53].

## 4. The Effect of Hypertonic Saline on Infection and Inflammation

HTS does improve mucociliary clearance and lung function in patients with CF but a number of studies have also explored the possibility that HTS may impact upon the inflammatory response within the airways and in particular levels of the proinflammatory neutrophil chemokine, interleukin(IL)-8. In the long-term controlled trial of inhaled HTS in patients with CF performed by Elkins and colleagues (2006), measurements of the proinflammatory cytokines IL-6, IL-8, IL-10, and tumour necrosis factor-alpha (TNF-alpha) were made in sputum at the time of screening and several later points until 48 weeks after the implementation of HTS nebulisation [24]. However, no significant difference was found in sputum levels between these groups (IL-6, *P* = 0.94; IL-8, *P* = 0.36; IL-10, *P* = 0.81, and TNF-alpha, *P* = 0.38), although all samples tested were from the postrandomisation period and no direct comparison was made between pre- and postnebulisation sputum samples or from samples taken before starting HTS treatment. A second study by Aitken et al., (2003) measured IL-8 and neutrophil numbers in CF sputum at 5 sequential time points during the 20 minutes after HTS nebulisation. Although the percentage of neutrophils decreased (89 ± 5% to 86 ± 4%; *P* = 0.03), the concentration of IL-8 remained the same [54]. The beneficial effect of HTS on reducing airway neutrophil numbers was also observed in an animal model of induced lung injury, whereupon 7.5% HTS significantly reduced cell counts in bronchial lavage fluid from 46.8 ± 4.4 × 10^3^ to 24.5 ± 5.9 × 10^3^ cells/mL (*P* < 0.05).

Unexpectedly, it has also been shown that HTS conditions may actually increase IL-8 production by CF gland cells via the NF-*κ*B pathway [55] and IL-8 expression in human bronchial epithelial cells via p38 mitogen-activated protein kinases activation [56]. Moreover, a study of individuals with asthma or COPD (10 patients in each group) revealed that inhalation of HTS caused low levels of inflammation in the airways with an increase in the levels of IL-6 and TNF-alpha recorded in exhaled breath condensate [57]. In contrast, on a cellular level, the beneficial effects of HTS were found to include reduced arachidonic acid and leukotriene-B_4_-induced priming of the respiratory burst of isolated neutrophils [58] and suppression of mTOR activity in mononuclear cells [59]. In additional studies, the beneficial effects of HTS were revealed as HTS increased levels of glutathione and thiocyanate which are protective against oxidants in the lung [60].

A further study by Suri et al., (2001) compared the effects of HTS and dornase-alpha on inflammatory mediators and found no significant difference in CF sputum IL-8 levels before and 18 hours after HTS nebulisation [17]. Indeed, it has been proposed that rhDNAse may actually promote inflammation by liberating cationic mediators bound to extracellular DNA such as proteases as well as active IL-8, which can potentiate neutrophilic inflammation causing further lung damage [61, 62]. This latter point is obviously a concern, but research by our group has shown that when IL-8 is released from negatively charged matrices including GAGs this renders the chemokine susceptible to proteolysis and that there is a period of time during which IL-8 levels are significantly decreased after HTS treatment [23]. As per results demonstrated by Frevert et al., (2003) [63], within our study, we found that IL-8 in CF bronchial lavage fluid is present in high molecular weight complexes involving GAGs including heparan and chondroitin sulphate [23]. By disrupting ionic interactions between IL-8 and GAGs, HTS displaced IL-8 from GAG matrices rendering the chemokine susceptible to proteolytic degradation by neutrophil elastase thereby impacting upon inflammation [23]. Another key inflammatory mediator that is found in the CF lung and thought to play an important role in the pathophysiology of CF lung disease is the antimicrobial peptide cathelicidin (LL-37) [64, 65]. LL-37 demonstrates antimicrobial activity against an array of bacteria including *S. aureus*, *Escherichia coli* [66], and *P. aeruginosa *[21] and although present in high concentrations within the CF lung, the activity of LL-37 is inhibited by binding to GAGs [66]. Release of LL-37 within CF BALF was brought about by enzymatic digestion of GAGs (by hyaluronidase, chondroitinase ABC, or heparinase II) thereby increasing the bactericidal efficiency of CF BALF against *Pseudomonas* and *Staphylococcus *bacteria [21]. In turn, HTS may also improve lung function by disrupting electrostatic interactions between GAGs and antimicrobial peptides. In support of this theory, *in vivo* LL-37 in CF sputum was liberated from GAGs following nebulised HTS (7%) resulting in increased antimicrobial effect [21]. Thus, HTS therapy may directly impact upon the viability of bacteria within the CF airways. In fact, the effect of HTS on *P. aeruginosa *appears multifold, with high ionic strength affecting not only flagellin-mediated motility [22], but also viability of the mucoid subpopulation [67].

## 5. How Does Hypertonic Saline Compare with Other Treatments?

While HTS is cost effective [68] and has been proposed to enhance mucociliary clearance and lung function in CF, other mucus mobilising therapies that have been evaluated include dornase-alpha [69], inhaled mannitol [70], gelsolin that severs actin filaments [30] and thiol derivatives such as n-acetylcysteine, although the clinical benefits of the latter treatment are unclear [71]. As high concentrations of DNA contribute to the viscosity of airway secretions, treatment with dornase-alpha, an enzyme which cleaves DNA polymers, results in a significant decrease in the viscosity of mucopurulent sputum [72]. Randomised controlled trials have demonstrated an improvement in FEV1 in patients treated with dornase-alpha [73–75] and although it appears more effective than HTS, some variation in the individual patient response was evident [76]. A small randomised study of 14 patients by Ballmann and Von Der Hardt, showed a mean increase in FEV1 of 7.7% in those treated with HTS in comparison to 9.3% in individuals treated with dornase-alpha [15]. A larger study of 48 children randomised to 12 weeks of daily dornase-alpha (2.5 mg), alternate day dornase-alpha, and twice daily HTS (7%) demonstrated a mean increase in FEV1 of 16% (SD 25%), 14% (SD 22%), and 3% (SD 21%) in each of the treatment groups, respectively. These studies strongly suggest that dornase-alpha is more effective than HTS in this setting [17].

Inhaled mannitol therapy has been proposed as an additional strategy to improve the airway surface hydration and mucociliary clearance in patients with CF. Mannitol is an osmotic agent with a high molecular weight which improves airway surface hydration by slow influx of water through a pericellular pathway [70]. Earlier studies have shown that mannitol improved mucociliary clearance in patients with asthma and non-CF-related bronchiectasis [77, 78]. A two-week course of inhaled mannitol in patients with CF resulted in an increase in mean FEV1 from baseline of 7% (95% confidence interval, 3.3 to 10.7) as well as mean FEF_25–75_ by 15.5% (95% confidence interval, −6.5 to 24.6) compared with placebo [79]. While an international trial assessing the effect of inhaled dry powder mannitol on lung function in CF showed a sustained clinical benefit of mannitol irrespective of rhDNase [80], there have been no large randomised trials comparing the effect of HTS versus mannitol in the treatment of patients. A small study, however, comparing inhaled HTS and mannitol to placebo, showed that both HTS and mannitol demonstrated an improvement in bronchial mucus clearance in the postintervention period (8.7 ± 3.3% and 10.0 ± 2.3% resp.) [81].

## 6. Hypertonic Saline in Treatment of Other Airway Diseases

While the focus of this review has been on CF-related bronchiectasis, non-CF-related bronchiectasis is a common clinical condition and is being recognised much more frequently with a reported prevalence in the United States of over 110,000. Whereas several studies have demonstrated benefits of HTS in CF, there are preliminary data regarding the therapeutic benefits of HTS in patients with non-CF bronchiectasis that are relevant to this review. In a double-blinded study of 96 infants receiving repeated doses of nebulised 3% HTS or isotonic saline, those treated with HTS had a clinically relevant 26% reduction in hospital length of stay [82]. In a small study of 24 individuals with stable non-CF bronchiectasis, Kellett and colleagues (2005) reported that patients treated with 7% HTS showed significantly higher sputum weights, reduced sputum viscosity, and ease of expectoration than those treated with isotonic saline (*P* < 0.0001). Median FEV1 and FVC demonstrated a statistically significant improvement (*P* = 0.043) [83]. In addition, a recent study by the same author reported that HTS was effective in decreasing sputum retention in patients with non-CF bronchiectasis, resulting in improved lung function and a reduction in annualised antibiotic usage and emergency health care utilisation [84].

Bronchiolitis is the leading cause of hospitalisation for infants involving a viral infection that begins as an upper-respiratory infection and then progresses to involve the lower small airways of the lung. A study by Al-Ansari et al., (2010) evaluated the clinical utility of HTS compared to isotonic saline in treatment of children with bronchiolitis and demonstrated that nebulisation with 5% HTS improved the bronchiolitis severity score in patients with viral bronchiolitis compared to treatment with 0.9% and 3% saline [85]. HTS has been also been suggested for use in chronic pulmonary disease (COPD), asthma, and pneumonia [86–90]. A study into the safety of sputum induction in 100 individuals with COPD reported that HTS could be used by tailoring treatment to a patient's specific needs in moderate-to-severe COPD [90]. Moreover, in a multicenter study of 79 subjects with moderate-to-severe asthma, indices of inflammation (IL-8, myeloperoxidase, eosinophilic cationic protein, and neutrophil elastase) in HTS-induced sputum were as reproducible as those present in sputum postmethacholine PC_20_ treatment [87].

## 7. Conclusion

Therapies acting against airway mucus in CF include dextran, nacystelyn [91], and gelsolin [30] which possess mucolytic properties, but evidence that these agents cause sustained relief from airway obstruction in chronic lung disease is lacking. On comparing the beneficial effects of aerosol administration of dornase-alpha to HTS [17], dornase-alpha improved FEV1 and reduced the frequency of pulmonary exacerbations [73] but illustrated a combined effect when administered with HTS for the clearance of CF purulent sputa [18]. HTS treatment is associated with an improvement in lung function and marked benefits with respect to exacerbations (Table 1) [24, 25, 92, 93]. As declared by Elkins and Bye (2006), “HTS appears broadly applicable as an inexpensive therapy for most patients with CF” [92] and of tremendous value on an individual basis for patients intolerant of dornase-alpha [12].

**Figure 4:**
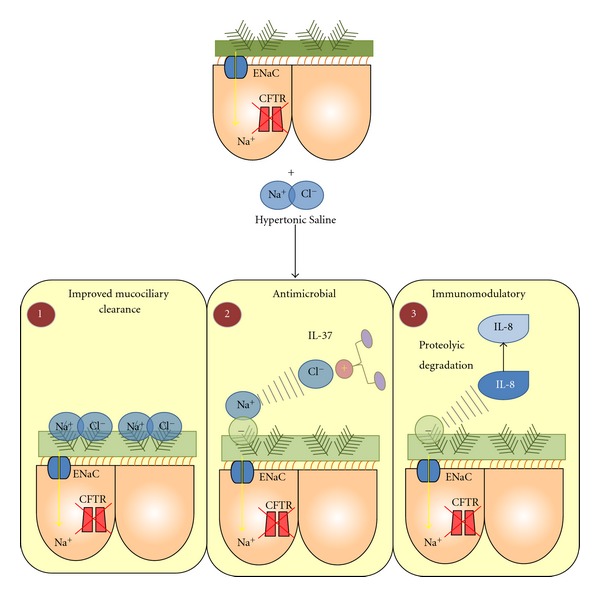
Schematic representation of the antimicrobial, immunomodulatory and mucolytic properties of HTS. (1) HTS draws water into the dehydated CF periciliary layer and improves mucus rheology and enhances mucociliary clearance. (2) LL-37, an antimicrobial protein that is inhibited by GAGs, is released by HTS via disruption of the electrostatic interaction between LL-37 and GAGs. (3) HTS liberates IL-8 from anionic matrices (GAGs) rendering the chemokine susceptible to proteolytic degradation by neutrophil elastase, thereby decreasing inflammation.

In a similar fashion to mannitol [94], the positive effect of nebulised HTS on mucociliary clearance is based on restoring the liquid layer lining the airways [26, 95]. This simple scheme, which for many years has served as a satisfactory working hypothesis, may not be the full story. Studies are now revealing that HTS can also function by releasing essential antimicrobial and immune molecules from complexation with ionic matrices thus improving both antimicrobial efficiency and resolution of inflammation (Figure 4). These observations suggest that HTS has beneficial therapeutic effects other than simply increasing mucociliary clearance and thus further investigations of the potential mechanisms of this currently available therapy is crucially required.

## Figures and Tables

**Table 1:** The reported effects of hypertonic saline on infection and inflammation.

HTS treatment	Patients sample or cells	Effect after HTS	Reference
7% HTS	Patients with CF	Higher FEV1 and FVC, less pulmonary exacerbations	Elkins et al. 2006 [24]
3% HTS	Sputum of patients with CF	Surfactant protein A increased; neutrophil counts, *Staphylococcus aureus* and non-mucoid *Pseudomonas* slightly decreased.	Aitken et al. 2003 [54]
Hypertonic medium	Human bronchial gland cells from CF and healthy controls (isolated from brushings)	Increased NaCl increased IL-8, but higher in CF cells (NF-*κ*B pathway activated)	Tabary et al. 2000 [55]
Hyperosmolarity (NaCl or mannitol, up to 6x normal)	Human bronchial epithelial cells	Increased IL-8 release via p38 and JNK pathway	Hashimoto et al. 1999 [56]
4.5% HTS	Exhaled breath condensate of patients with asthma or COPD and healthy controls	Greater IL-6 and TNF-alpha concentration, lower pH.	Carpagnano et al. 2005 [57]
Hypertonic medium	Peripheral blood neutrophils	HTS inhibited neutrophil priming of respiratory burst by LTB_4_ and arachidonic acid	Lee et al. 2011 [58]
Hypertonic medium	Peripheral blood mononuclear cells	Reduced LPS induced mTOR pathway activation in HTS treated cells	Schaeffer et al. 2010 [59]
7% HTS	Bronchial samples	Increased antioxidant levels in BAL fluid	Gould et al. 2010 [60]
7% HTS	Sputum from patients with CF	Decreased IL-8 concentration in sputum after HTS	Reeves et al. 2011 [23]
7% HTS	Sputum from patients with CF	LL-37 complexation to GAGs was decreased after HTS and antimicrobial properties of sputa restored	Bergsson et al. 2009 [21]
2–7% HTS in culture medium	*Pseudomonas* strain PA01 and mucoid strain FRD1	Reduced motility and growth of all strains tested	Havasi et al. 2008 [22]
0–0.8 M NaCl added to medium	*Pseudomonas* strain PA01 and *mucA* mutant	MucA mutant less resistant to osmotic stress	Behrends et al. 2010 [67]
